# Identification of CBX3 and ABCA5 as Putative Biomarkers for Tumor Stem Cells in Osteosarcoma

**DOI:** 10.1371/journal.pone.0041401

**Published:** 2012-08-03

**Authors:** Vaibhav Saini, Curtis D. Hose, Anne Monks, Kunio Nagashima, Bingnan Han, Dianne L. Newton, Angelena Millione, Jalpa Shah, Melinda G. Hollingshead, Karen M. Hite, Mark W. Burkett, Rene M. Delosh, Thomas E. Silvers, Dominic A. Scudiero, Robert H. Shoemaker

**Affiliations:** 1 Screening Technologies Branch, Developmental Therapeutics Program, Division of Cancer Treatment and Diagnosis, National Cancer Institute at Frederick, Frederick, Maryland, United States of America; 2 SAIC-Frederick, National Cancer Institute at Frederick, Frederick, Maryland, United States of America; 3 Biological Testing Branch, Division of Cancer Treatment and Diagnosis, National Cancer Institute at Frederick, Frederick, Maryland, United States of America; Inserm U606 and University Paris Diderot, France

## Abstract

Recently, there has been renewed interest in the role of tumor stem cells (TSCs) in tumorigenesis, chemoresistance, and relapse of malignant tumors including osteosarcoma. The potential exists to improve osteosarcoma treatment through characterization of TSCs and identification of therapeutic targets. Using transcriptome, proteome, immunophenotyping for cell-surface markers, and bioinformatic analyses, heterogeneous expression of previously reported TSC or osteosarcoma markers, such as CD133, nestin, POU5F1 (OCT3/4), NANOG, SOX2, and aldehyde dehydrogenase, among others, was observed *in vitro.* However, consistently significantly lower CD326, CD24, CD44, and higher ABCG2 expression in TSC-enriched as compared with un-enriched osteosarcoma cultures was observed. In addition, consistently higher CBX3 expression in TSC-enriched osteosarcoma cultures was identified. ABCA5 was identified as a putative biomarker of TSCs and/or osteosarcoma. Lastly, in a high-throughput screen we identified epigenetic (5-azacytidine), anti-microtubule (vincristine), and anti-telomerase (3,11-difluoro-6,8,13-trimethyl- 8H-quino [4,3,2-kl] acridinium methosulfate; RHPS4)-targeted therapeutic agents as candidates for TSC ablation in osteosarcoma.

## Introduction

Osteosarcoma is the second highest cause behind cancer-related deaths in the pediatric age group [1]. Despite multimodal chemotherapy, the mortality rate has not significantly improved since the 1970 s. Relapse observed after chemotherapy is associated with <20% survival [1], [2]. In recent years, tumor stem cells (TSCs) have been implicated in tumorigenesis and response to treatment of many tumor types [3]. Therefore, to improve osteosarcoma treatment, strategies to eradicate TSCs are needed. As a step towards identification of such strategies, TSCs in osteosarcoma need to be characterized and therapeutic targets need to be identified.

Recently, vitronectin in serum has been implicated in promoting differentiation of TSCs of breast and prostate cancers in culture [4]. Tumor cell lines and patient samples cultured in serum-free, growth factor-supplemented conditions have been reported to form spheres. These spheres have been shown to be enriched for TSCs [3]. This maneuver has been used to derive spheres from osteosarcoma cell lines, such as Saos-2 and MG-63 [5], [6].

TSC-enrichment by sphere culture can be monitored by analyzing the expression of various TSC-implicated markers, such as ATP-binding cassette (ABC) transporters, aldehyde dehydrogenases (ALDHs), CD133, POU5F1 (OCT3/4), SOX2, and NANOG. ABC transporters have been reported to confer chemoresistance on tumors and TSCs [7], [8]. In particular, ABCG2 expression has been used to identify a drug-resistant side population or TSCs in a variety of tumors, such as pulmonary, liver, pancreatic, colon tumors, and osteosarcoma [9]. ALDHs have been reported to function in endobiotic and xenobiotic metabolism. ALDHs function in the metabolism of retinoic acid, alcohol, cyclophosphamide, aldehydes produced during lipid metabolism, and oxidative stress [10]. ALDH has been reported as a marker for TSCs in a variety of tumors, such as breast cancer, leukemia, and osteosarcoma [6], [11], [12]. CD133 has been used to identify TSCs from breast, lung, liver, colon, prostate, brain, and bone cancers [13], [14], [15], [16], [17].

Expression of POU5F1 (OCT3/4), SOX2, and NANOG has been used to identify TSCs in pulmonary neoplasms, oral squamous cell carcinoma, and glioblastoma [18], [19], [20]. POU5F1 promoter driven GFP expression in a transiently transfected cell line derived from an osteosarcoma biopsy has been used to identify tumorigenic cells [21] However, in another study, expression of POU5F1, SOX2, and NANOG was reported to either increase significantly or remain unchanged in sphere cultures as compared with non-TSC enriched cultures of Os 99-1, Saos-2, MG-63, and HuO9 osteosacroma cell lines [5]. Therefore, identification of TSCs based on POU5F1, SOX2, or NANOG expression remains controversial, at least in osteosarcoma.

Taken together, several different proteins have been proposed to identify TSCs in osteosarcoma. These studies depended on the use of previously published SC markers. To identify novel putative TSC markers, we performed genome- and proteome-wide analyses, cell surface marker immunoprobing, and bioinformatic evaluation of spheres derived from osteosarcoma cells. Further, we performed high-throughput *in vitro* drug sensitivity phenotyping to identify potential intervention opportunities for ablation of TSC-enriched osteosarcoma cultures.

## Results

### Sphere culture enriched for self-renewing, clonogenic, and tumorigenic cells

*CHA59 clinical isolate:* Surgery was performed on a previously untreated 16-year-old male patient with osteosarcoma of the distal femur at the Children's Hospital Medical Center, Akron Ohio, USA. The primary tumor sample was used (by R.H.S.) to establish the initial CHA59 culture. Subsequently the CHA59 cell line was deposited at the NCI tumor repository. CHA59 stained positive for alkaline phosphatase (ALPL), and treatment with osteoblastic differentiation cocktail increased the intensity of ALPL staining (Figure S1A). CHA59 xenografts established in NOD/SCID mice showed osteoid production (Figure S1B). CHA59 spectral karyotyping showed multiple chromosomal abnormalities (Figure S1C). Saos-2 and HuO9 are well-documented osteosarcoma cell lines with an osteoblastic phenotype. Saos-2 has been reported to express ALPL and produce mineralized matrix [22], [23]. Similarly, HuO9 has been reported to express ALPL and produce osteoid and mineralized matrix *in vivo* [24].

**Figure 1:**
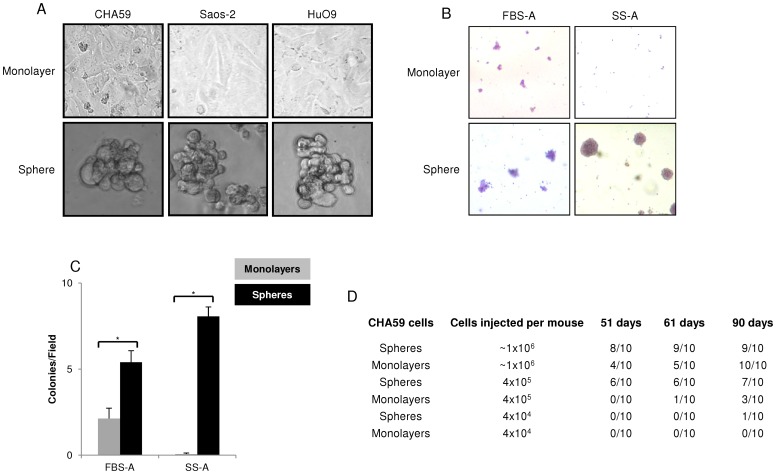
Spheres demonstrated increased clonogenicity and tumorigenicity compared with monolayers. (A) CHA59, Saos-2, and HuO9 monolayers generated spheres in FBS-free, SS media. Phase contrast images taken with a 40× objective. (B) CHA59 spheres formed colonies in both FBS-A (Fetal Bovine Serum-Agarose) and SS-A (KnockOut Serum Replacement-Agarose). Images taken with a 10× objective. C) CHA59 spheres demonstrated significantly higher clonogenicity as compared with monolayers in FBS-A and SS-A, * p<0.05. (D) CHA59 Spheres demonstrated higher tumorigenicity than monolayers. 10 NOD/SCID mice injected with cells per group. A xenograft of ≥150 mg was counted positive for take rate.

CHA59, Saos-2, and HuO9 cells grew as monolayers in medium containing FBS (Figure 1A). In serum-free, growth factor supplemented medium (SS), a morphological change from monolayers to spheres was observed (Figure 1A). Next, monolayers and spheres were analyzed for their clonogenic ability, an established *in vitro* functional TSC correlate. Whereas monolayers formed colonies only on agarose that contained FBS and none on agarose that contained SS (Figure 1B and S1D), spheres demonstrated colony-forming ability in either matrix. Moreover, as compared with monolayers, spheres formed significantly more colonies in agarose that contained either FBS or SS (Figure 1C).

Besides clonogenicity, spheres demonstrated self-renewal ability as tested by the production of subsequent sphere generations (Figure S1E).

*In vivo* tumorigenicity of CHA59 monolayers and spheres was compared in immunocompromised NOD/SCID mice with matrigel support of injected cells (Figure 1D). At an input of 4×10^4^ cells per mouse, tumor formation was observed in only 1 mouse injected with spheres after 90 days. At an input of 4×10^5^ cells per mouse, a consistently higher tumorigenic ability was observed for spheres than monolayers at 51, 61, and 90 days post injection. Further, at an input of ≥1×10^6^ cells per mouse, more tumors were observed for spheres than monolayers at 51 and 61 days; both the samples generated tumors at 90 days. Thus, sphere culture was enriched for clonogenic and tumor-initiating cell populations.

### Spheres contained a heterogeneous cell population

**Figure 2:**
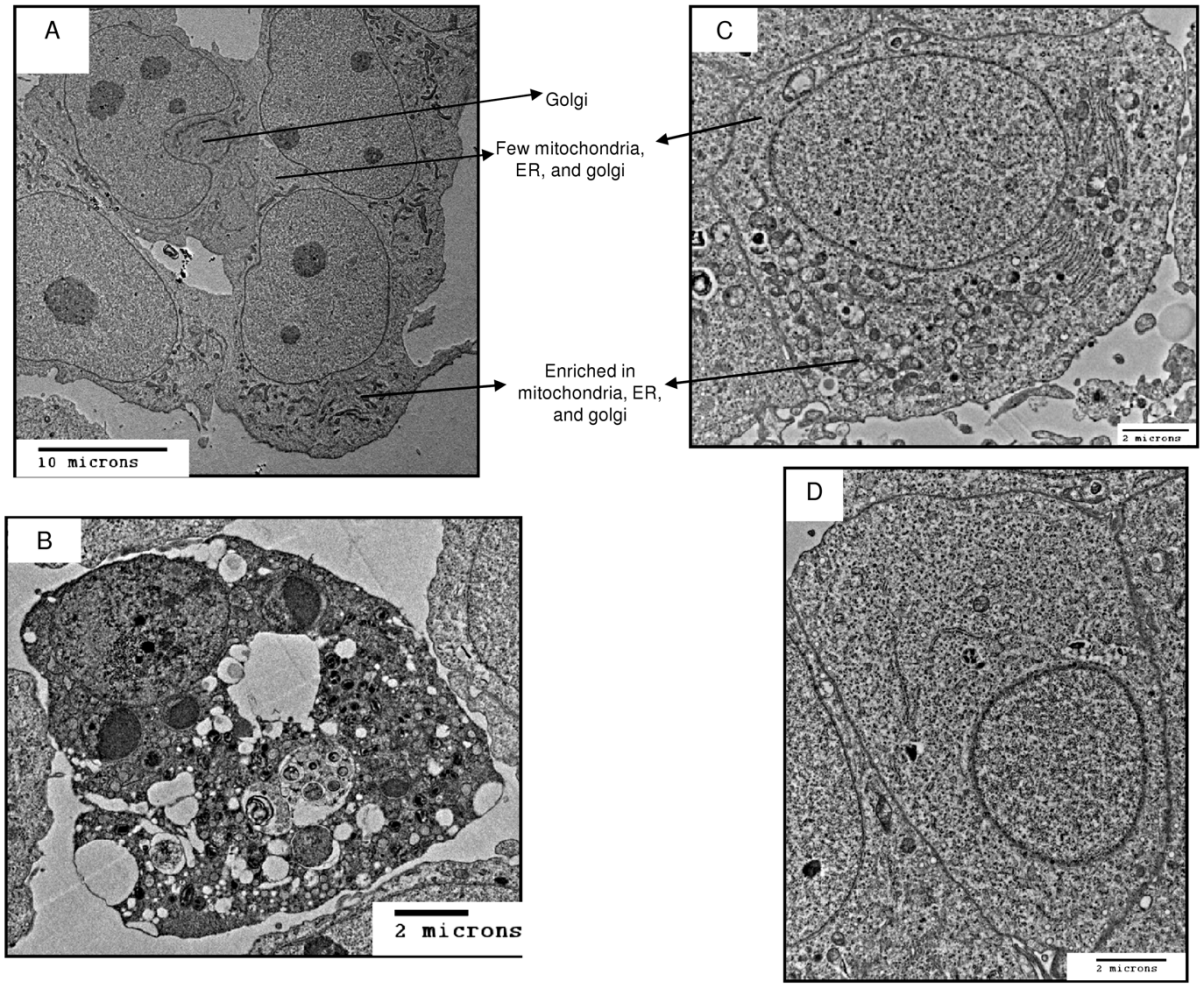
CHA59 spheres contained a heterogeneous cell population as compared with monolayers, visualized by transmission electron microscopy. (A) Monolayers contained polarized cells – one end rich in organelles, 500× magnification. (B, C, D) Spheres contained (B) dead (1,000× magnification), (C) polarized (2,000× magnification), and (D) organelle-deficient cells (2,000× magnification).

The ultrastructural features of CHA59 monolayers and spheres were characterized by transmission electron microscopy. The monolayers contained a relatively homogeneous cell population with abundant cytoskeletal elements, golgi apparatus, normal mitochondria and endoplasmic reticulum, and polarized cytoplasm (Figure 2A). In contrast, spheres displayed a heterogeneous population consisting of dead cells (Figure 2B); polarized cells (Figure 2C), similar to those observed in monolayers; and cells devoid of organelles except for a nucleus (Figure 2D).

### Migration and invasion phenotype of spheres

CHA59 monolayers and spheres demonstrated substantial invasion ability towards FBS in the transwell assay as compared with BSA control (Figure S2A). However, spheres showed significantly reduced invasion towards FBS as compared with monolayers (Figure S2A). Next, we analyzed the invasion ability of spheres towards FBS and SS chemoattractant. In a 48-hour manual invasion assay, spheres showed significantly higher invasion towards FBS as compared with either BSA or SS (Figure S2B).

To further analyze the migration and invasion phenotype of monolayers and spheres, a 7-day real-time kinetic assay was employed. HuO9 spheres demonstrated significantly higher (p<0.05) migration and invasion ability than monolayers towards the SS chemoattractant (Figure S2C).

Saos-2 spheres showed significantly lower migration ability than monolayers towards the FBS and SS chemoattractant (Figure S2D). Moreover, significantly higher migration ability was recorded for both monolayers and spheres towards the FBS than the SS chemoattractant. However, sphere-formation was only observed for cells that migrated towards the SS (Figure S2E, bottom row) and not the FBS chemoattractant (Figure S2E, top row). For this purpose, at the end of the assay, the migrated cells were collected from the bottom chamber. Cells from both monolayers and spheres that migrated towards the SS chemoattractant formed spheres (Figure S2E, bottom row). On the contrary, cells from both monolayers and spheres that migrated towards the FBS chemoattractant remained as individual cells (Figure S2E, top row). Thus, this demonstrates that the cells that migrated towards the SS medium formed spheres.

The trend for higher invasion ability of CHA59 monolayers compared to spheres towards FBS in the 48-hour manual assay is similar to that observed for Saos-2 and HuO9 in the 7-day kinetic assay (Figure S2A–D). Further, the low CHA59 sphere invasion ability towards SS in the 48-hour manual assay corroborates with the 7-day kinetic migration/invasion signal recorded for Saos-2 and HuO9, which showed signal after 5 days and almost none at 48 hours (Figure S2A–D).

### Sphere gene signature associated with stem cell pathway

Gene expression pattern analysis in CHA59 cells identified 772 genes as significantly (p<0.01) modulated >2-fold in spheres vs. monolayers. In addition, Ingenuity Pathway analysis (Ingenuity Systems Inc.; https://ingenuity.analysis.com) revealed that the canonical pathway most significantly associated with these data was the Human Embryonic Stem Cell Pluripotency Pathway (p = 9×10^−7^). Out of the differentially modulated genes, those implicated in stem cell and osteosarcoma biology, such as PPARG, ETS1, WNT1, WNT5B, SOX2, NANOG, POU5F1, nestin, and ALDH were chosen for further analysis.

**Figure 3:**
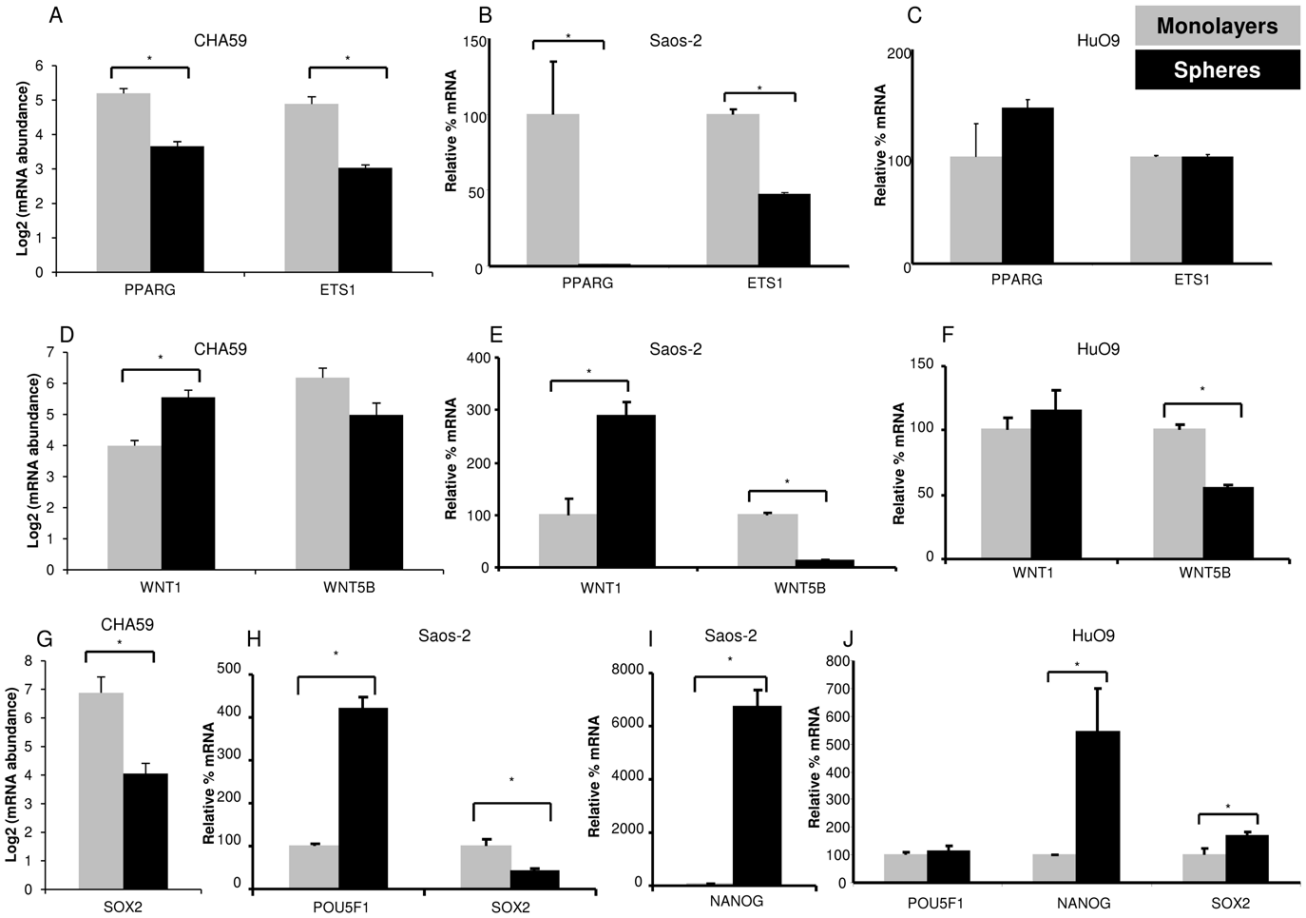
Expression of transcription factors and signaling molecules in CHA59, Saos-2, and HuO9 monolayers and spheres. (A, D, G) CHA59 cells, transcript abundance measured by microarray analysis, (B, E, H, I) Saos-2 cells, transcript level measured by real-time RT-PCR. (C, F, J) HuO9 cells, transcript level measured by real-time RT-PCR. *p<0.05.

PPARG and ETS1 were expressed at lower levels in CHA59 spheres than monolayers (Figure 3A). Saos-2 cells demonstrated the same trend (Figure 3B). HuO9 monolayers and spheres expressed comparable PPARG and ETS1 levels (Figure 3C).

WNT1 expression was significantly higher and WNT5B was expressed at comparable levels in CHA59 spheres and monolayers (Figure 3D). Saos-2 spheres demonstrated significantly higher WNT1 and lower WNT5B levels than monolayers (Figure 3E). HuO9 monolayers and spheres expressed WNT1 at comparable levels; however, spheres displayed a significantly lower WNT5B expression than monolayers (Figure 3F).

SOX2 transcript was significantly lower in CHA59 spheres than monolayers (Figure 3G), and no differences were observed for POU5F1 and NANOG (data not shown). Saos-2 spheres displayed significantly higher POU5F1 and NANOG and lower SOX2 levels than monolayers (Figure 3H, I), as reported by another laboratory as well [5]. HuO9 monolayers and spheres expressed POU5F1 at comparable levels; however, spheres contained significantly higher NANOG and SOX2 than monolayers (Figure 3J).

### Differential expression of cytoskeletal and nuclear proteins in monolayers and spheres

**Figure 4:**
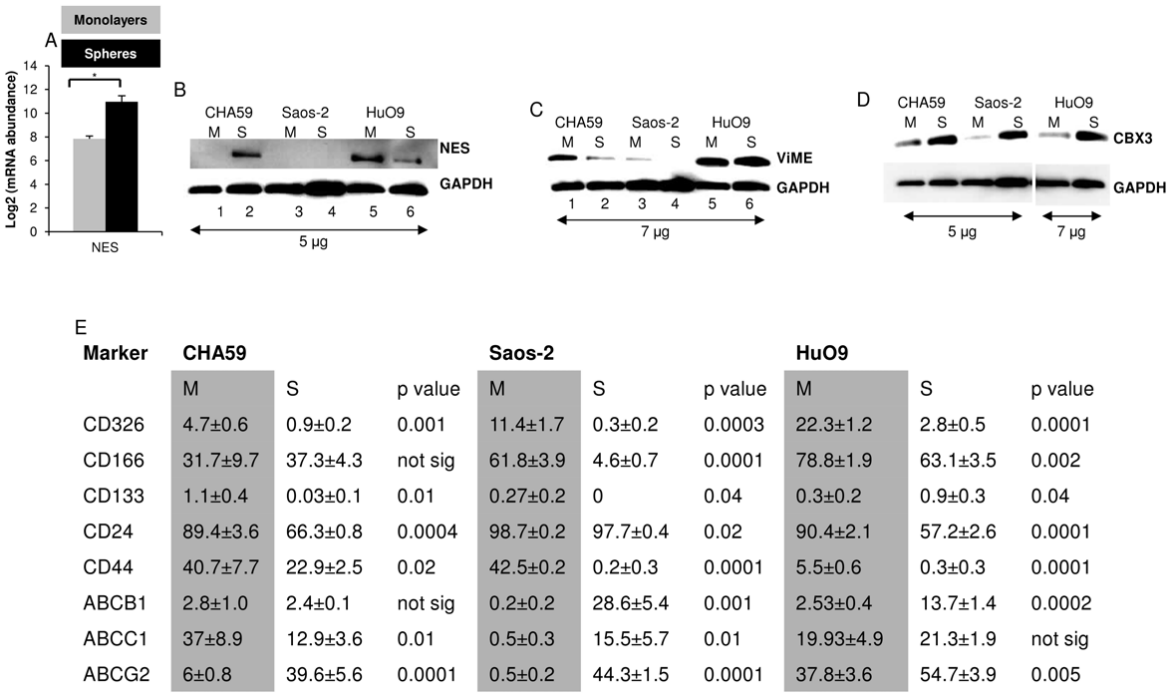
Differential transcript and protein expression in monolayers and spheres. (A) Nestin (NES) transcript in CHA59 monolayers and spheres measured by microarray analysis, * p<0.05. (B) Nestin (NES), (C) vimetin (VIME), and (D) chromobox protein homolog 3 (CBX3) protein expression in CHA59, Saos-2, and HuO9 cells tested by western blotting. ‘M’ represents monolayers, ‘S’ represents spheres. (E) Immunophenotype of monolayers and spheres probed by flow cytometry. CHA59 and Saos-2 immunophenotype reported as a cumulative of 6-color panel (CD326, CD166, CD133, CD24, CD44, excludes ABCB1) and 3-color panel (ABCB1, ABCC1, ABCG2). HuO9 immunophenotype reported as a cumulative of 6-color panel (CD326, CD166, CD133, CD24, CD44, ABCB1) and 2-color panel (ABCC1, ABCG2). p value calculated for monolayers vs. spheres for each cell line. ‘M’ represents monolayers, ‘S’ represents spheres.

Proteomic analysis of CHA59 monolayers and spheres identified differential expression of cytoskeletal elements and nuclear proteins (Figure S3). Translationally controlled tumor protein, chromobox protein homolog 3 (CBX3), malate dehydrogenase, dihydropyrimidinase-related protein 2, and fructose-bisphosphate aldolase C levels were higher, and vimentin (VIME) and median-chain specific acyl-CoA dehydrogenase levels were lower in CHA59 spheres than monolayers (Figure S3). VIME, previously reported as a marker for mesenchymal stem cell lineage [25], and CBX3, previously reported to be upregulated in tumors [26], were chosen for further analysis. VIME expression was higher in CHA59 and Saos-2 monolayers than spheres (Figure 4C, lane 1, 2, 3, 4), but it was expressed at comparable levels in HuO9 monolayers and spheres (Figure 4C, lane 5, 6). CBX3 expression was consistently higher in spheres than monolayers for the 3 cell lines (Figure 4D).

Nestin transcript showed a significantly higher expression in CHA59 spheres than monolayers (Figure 4A), and was confirmed at the protein level (Figure 4B, lane 1, 2). It was undetectable in Saos-2 (Figure 4B, lane 3, 4). Its expression was reversed in HuO9 monolayers and spheres (Figure 4B, lane 5, 6).

### ABC transporter phenotype of monolayers and spheres

CHA59, Saos-2, and HuO9 were immunoprobed for the expression of cell surface markers (CD133, CD24, CD166, CD326, CD44, ABCB1, ABCC1, ABCG2) previously reported to be associated with TSCs and/or osteosarcoma [3], [17], [27]. The absolute expression levels of the markers varied among the 3 cell lines. Despite this, the marker profile (Figure 4E) revealed the following patterns: 1. CD326, CD24, and CD44 decreased significantly in spheres compared with monolayers for the 3 cell lines, 2. CD166 either remained the same (CHA59) or decreased significantly in spheres than monolayers, and the decrease was more dramatic in Saos-2 spheres than HuO9 spheres, 3. CD133 levels significantly decreased in CHA59 and Saos-2 spheres and increased in HuO9 spheres as compared with monolayers, 4. ABCB1 expression increased significantly in Saos-2 and Huo9 spheres compared with monolayers, but remained at comparable levels in CHA59, 5. ABCC1 levels significantly decreased in CHA59 spheres compared with monolayers, significantly increased in Saos-2 spheres compared with monolayers, and remained about the same in HuO9 spheres and monolayers, 6. ABCG2 demonstrated a significant increase in spheres compared with monolayers for the 3 cell lines, and 7. Out of the 3 cell lines, only Saos-2 spheres demonstrated a significantly higher expression of the 3 ABC transporters compared with monolayers.

Transcriptome data showed significantly higher ABCA5 expression in CHA59 spheres compared with monolayers (Figure S4A). Saos-2 paralleled the trend (Figure S4B). HuO9 expressed ABCA5 at comparable levels in monolayers and spheres (Figure S4C).

ABCB1, ABCC1, and ABCG2 expression was measured in CHA59 and Saos-2 in a 3-color panel, and thus, only these data were used for bioinformatic analyses. Based on the ABCB1, ABCC1, and ABCG2 expression, CHA59 and Saos-2 monolayers and spheres produced 10 clusters (C1–10) (Figure S5A). There were triple positive ABCB1/ABCC1/ABCG2 (C2 and C7), double positive ABCB1/ABCC1 (C10), and single positive ABCG2 (C4, C6, and C9) clusters, but there were no single positive ABCB1 or ABCC1 clusters. For CHA59, the population in ABCG2 positive C3 increased in spheres (14%) compared with monolayers (5%) (Figure S5B). Triple positive C2 cells with average size showed similar population distribution (5%) in CHA59 monolayers and spheres (Figure S5B). Interestingly, the population in triple positive C2 increased in Saos-2 spheres (17%) compared with monolayers (0%). Further, principal component analysis of the color-coded clusters distinguished the CHA59 and Saos-2 monolayers and spheres (Figure S5C).

### Aldehyde dehydrogenase and ABC transporter expression in monolayers and spheres

**Figure 5:**
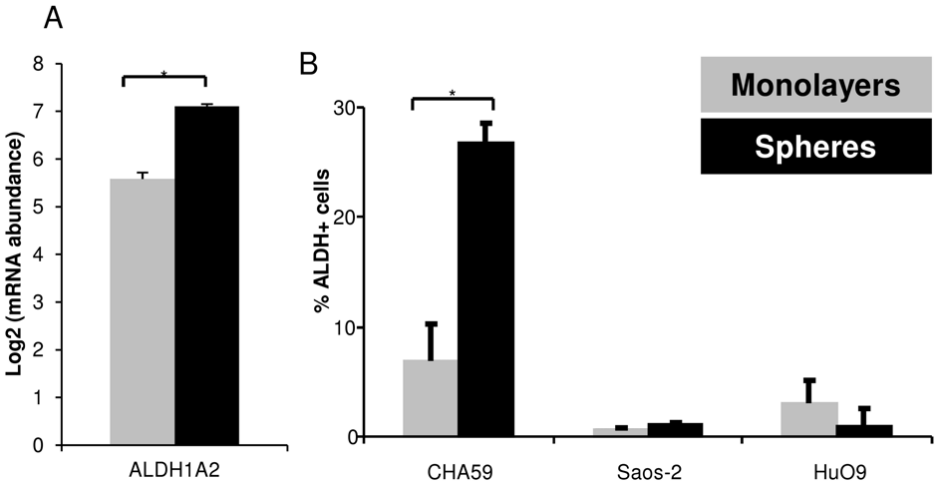
Heterogeneous aldehyde dehydrogenase (ALDH) expression in monolayers vs. spheres. (A) Aldehyde dehydrogenase 1 family, member A2 (ALDH1A2) transcript in CHA59 monolayers (gray bar) and spheres (black bar) measured by microarray analysis, * p<0.05. (B) Percentage of ALDH expressing cell population in CHA59, Saos-2, and HuO9 monolayers and spheres measured by ALDEFLUOR assay. *p<0.05.

ALDH1A2 transcript was expressed at a significantly higher level (p = 0.0013) in CHA59 spheres than monolayers (Figure 5A). This ALDH expression pattern was confirmed by FACS analysis (Figure 5B). However, a negligible amount of ALDH was observed in the Saos-2 and HuO9 cells (Figure 5B).

**Figure 6:**
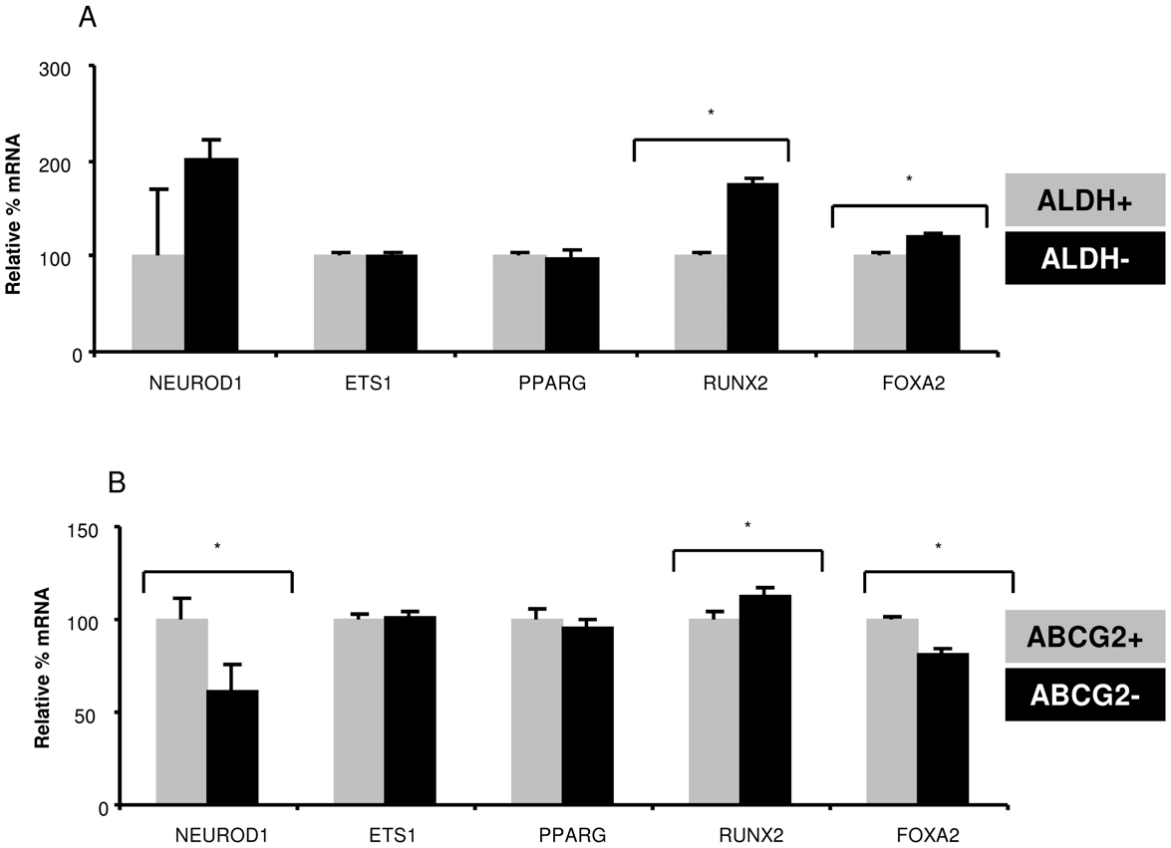
Differential profile of stem cell biology-implicated transcription factors in ALDH+/− and ABCG2+/− cells sorted from CHA59 spheres. (A) ALDH+/− subpopulations were sorted from CHA59 spheres using ALDEFLUOR staining and FACS. Subsequently, expression of various genes was measured in the sorted population by real-time RT-PCR analysis. *p<0.05. (B) ABCG2+/− subpopulations were sorted from CHA59 spheres using an anti-ABCG2 antibody and FACS. Thereafter, expression of various genes was measured in the sorted population by real-time RT-PCR analysis. *p<0.05.

To understand if ALDH and ABCG2 expression marked the same or distinct cell populations, CHA59 spheres were sorted into ALDH+/− and ABCG2+/− subpopulations. These were characterized for the expression of various transcription factors implicated in tumorigenesis and/or osteosarcoma [28], [29], [30], [31], [32]. ALDH+ cells showed significantly lower RUNX2 and FOXA2, and comparable NEUROD1, ETS1, and PPARG expression than ALDH− cells (Figure 6A). ABCG2+ cells displayed significantly higher NEUROD1 and FOXA2, lower RUNX2, and comparable ETS1 and PPARG expression than ABCG2- cells (Figure 6B).

**Figure 7:**
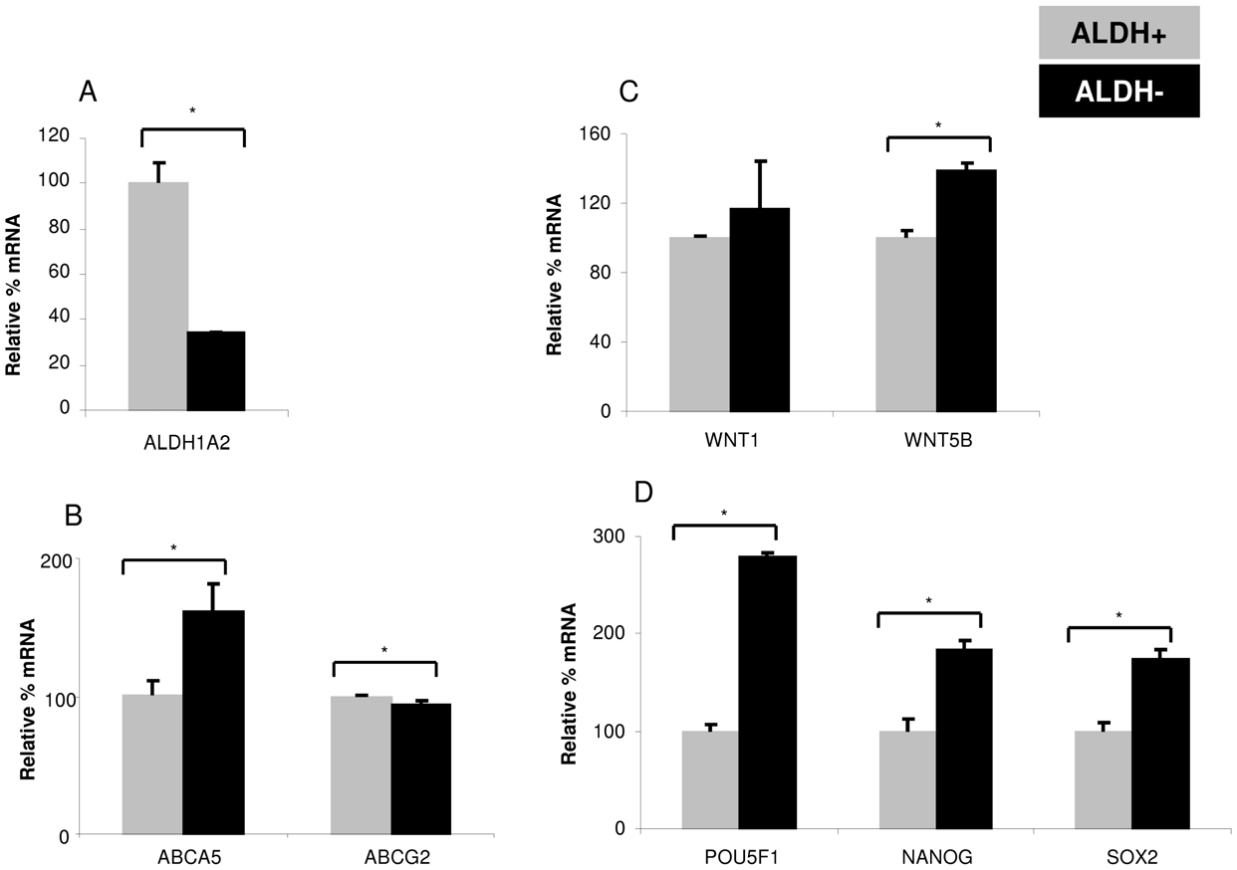
Differential transcript profile of ALDH+ and ALDH− subpopulations sorted from CHA59 spheres. (A–D) ALDH+/− cells were sorted from CHA59 spheres using ALDEFLUOR staining and FACS. Subsequently, expression of various genes was measured in the sorted population by real-time RT-PCR analysis. *p<0.05.

To probe an overlap of ABC transporter and ALDH expression in sorted subpopulations, ALDH+/− cells were analyzed further. As expected, ALDH+ cells showed significantly higher ALDH1A2 transcript than ALDH− cells (Figure 7A). ALDH+ cells expressed significantly lower ABCA5 and higher ABCG2 than ALDH− cells (Figure 7B). Significantly higher WNT5B, POU5F1, NANOG, and SOX2, and comparable WNT1 transcripts were observed in ALDH− than ALDH+ cells (Figure 7C, 7D).

### CBX3 and ABCA5 expression in osteosarcoma patient biopsies

**Figure 8:**
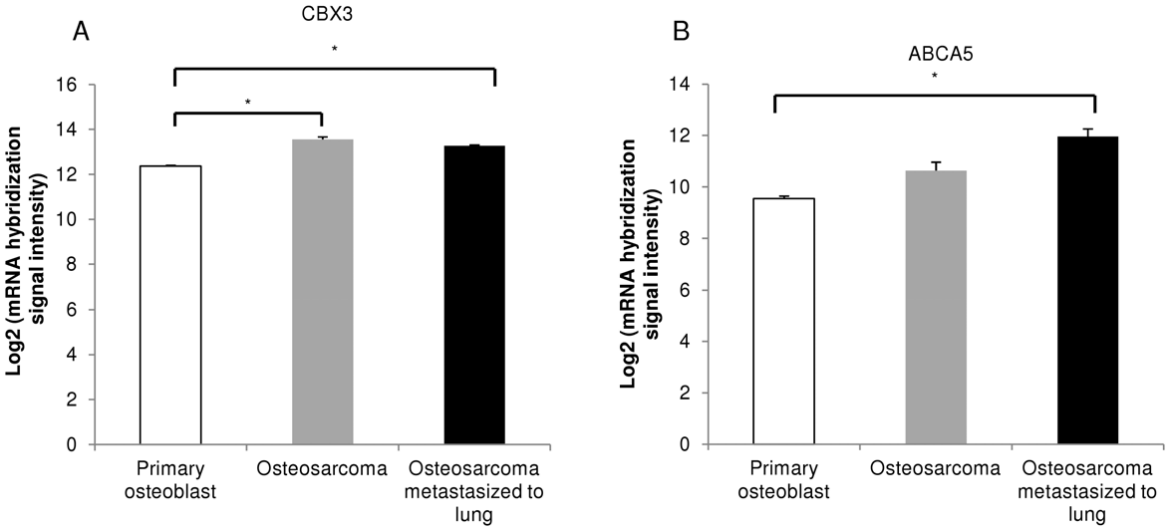
CBX3 and ABCA5 transcript expression in osteosarcoma biopsies. (A) CBX3 and (B) ABCA5 expression in osteosarcoma and osteosarcoma metastasized to lung biopsies as compared with primary osteoblasts, analyzed *via* microarray data available in GEO. *p<0.05.

To assess the expression of CBX3 and ABCA5 in patients, transcriptome data from osteosarcoma biopsies and primary osteoblasts, available from GEO, were analyzed. CBX3 expression was significantly higher in both osteosarcoma and osteosarcoma metastasized to lung as compared with primary osteoblasts (Figure 8A). ABCA5 expression was comparable between osteosarcoma and primary osteoblasts, but osteosarcoma metastasized to lung showed significantly higher ABCA5 expression when compared with primary osteoblasts (Figure 8B). For both CBX3 and ABCA5, comparable expression was observed between osteosarcoma and osteosarcoma metastasized to lung.

### Differential drug sensitivity of monolayers and spheres

In Table 1, spheres and monolayers demonstrated comparable sensitivity to approved drugs, such as cisplatin, 5-fluorouracil, and gleevac (imatinib), raising the possibility of modifying already existing therapeutic regimens to target both tumor cells and TSCs. Spheres, as expected, demonstrated higher resistance, when compared with monolayers, to compounds in a panel of anti-neoplastic agents, including clinically approved drugs, such as velcade, dasatinib, and sorefinib. However, CHA59 and Saos-2 spheres were more sensitive than monolayers to 5-azacytidine (5-aza-C). Saos-2 and HuO9 spheres, as compared with monolayers, were more sensitive to vincristine and RHPS4. Therefore, similar to the heterogeneous gene and protein expression profile, heterogeneous drug sensitivity profiles were observed for CHA59, Saos-2, and HuO9 cells.

**Table 1:** Differential drug resistance of CHA59, Saos-2, and HuO9 monolayer vs. sphere.

Small Molecule	NSC	CHA59		Saos-2		HuO9	
		M	S	M	S	M	S
Adriamycin	123127	1.3E–07	1.5E–07	1.4E–07	4.9E–08	8.0E–08	4.9E–08
Paclitaxel	125973	8.3E–08	4.5E–08	2.4E–08	7.7E–09	2.2E–08	1.8E–08
Topotecan	609699	2.4E–08	2.1E–08	3.5E–08	5.1E–09	1.5E–08	2.0E–08
Thiosemicarbazone derivative	73306	1.1E–05	>1.0E–04	6.8E–05	>1.0E–04	2.5E–05	>1.0E–04
17-DMAG	707545	7.6E–08	3.6E–07	1.8E–07	5.9E–08	2.6E–08	3.7E–08
Sterol mesylate	67657	3.8E–04	>1.0E–03	2.2E–04	>1.0E–03	1.8E–04	8.6E–04
Velcade	681239	1.0E–08	3.7E–08	1.5E–08	4.2E–08	5.0E–09	1.9E–08
Vincristine	67574	1.0E–07	1.4E–07	3.1E–08	4.3E–09	1.4E–07	1.5E–08
SU11274	747693	1.9E–05	>1.0E–04	>1.0E–04	>1.0E–04	>1.0E–04	>1.0E–04
Cisplatin	119875	>1.0E–04	>1.0E–04	>1.0E–04	>1.0E–04	>1.0E–04	>1.0E–04
5-fluorouracil	19893	8.7E–05	2.2E–05	1.1E–05	1.9E–05	>1.0E–04	>1.0E–04
5-azacytidine	102816	6.2E–06	6.2E–07	2.4E–05	4.3E–06	4.5E–05	3.2E–05
RHPS4	714187	2.4E–05	>1.0E–04	3.7E–05	8.5E–06	1.9E–05	4.1E–06
Sorefinib	747971	1.2E–05	3.7E–05	2.1E–05	3.9E–05	2.1E–05	3.9E–05
Dasatinib	732517	7.2E–07	2.2E–06	3.6E–06	1.8E–07	5.0E–08	4.8E–06
Imatinib	743414	2.6E–05	4.4E–05	3.2E–05	4.2E–05	2.6E–05	4.0E–05
PV1019	744039	9.8E–06	3.8E–05	1.4E–05	2.5E–05	1.6E–05	2.5E–05

Gray background marks higher drug sensitivity as measured by IC50 in molarity. ‘M’ represents monolayers, ‘S’ represents spheres. 17-DMAG (17-(Dimethylaminoethylamino)-17-demethoxygeldanamycin); SU11274 ([(3*Z*)-*N*-(3-chlorophenyl)-3-({3,5-dimethyl-4-[(4-methylpiperazin-1-yl)carbonyl]-1*H*-pyrrol-2-yl}methylene)-*N*-methyl-2-oxoindoline-5-sulfonamide]); RHPS4 (3,11-difluoro-6,8,13-trimethyl- 8H-quino [4,3,2-kl] acridinium methosulfate); PV1019 ([7-nitro-1*H*-indole-2-carboxylic acid {4-[1-(guanidinohydrazone)-ethyl]-phenyl}-amide]).

## Discussion

The primary objectives of our research were to characterize TSC-enriched sphere cultures, discover novel putative TSC and tumor markers in osteosarcoma, and identify potential therapeutic opportunities. Sphere culture enriched for clonogenic and tumorigenic cells. The tumorigenicity observed for CHA59 points to the rarity of TSCs in this cell line. In this regard, a range of tumorigenic abilities has been reported for osteosarcoma. For example, an input of 50 cells to ∼10,000 cells per mouse, depending on the cell line and mouse model, has been reported to form xenografts [11], [17], [33].

Organelle-devoid cells, termed Small Light Cells, have been reported as stem cells in rat and mouse mammary glands [34]. We observed organelle-devoid cells in spheres and none in monolayers, thereby confirming – taking into account the enhanced clonogenicity, tumorigenicity, and drug sensitivity of spheres as compared with monolayers – the ability of *in vitro* sphere culture to model *in vivo* TSC features.

The invasion ability of cells towards growth factor supplemented medium has been used to isolate sphere forming cells in prostate tumor cells [35]. In this report, we provide the first direct evidence, to our knowledge, that the cells that migrate towards growth factor supplemented medium form spheres. While we have not analyzed these spheres, other reports in the literature have shown that for prostate tumors transwell assay can be used to isolate cells that upon subculture form spheres, and that the TSC marker pattern is the same for invaded cells and those isolated from spheres cultured in serum-free supplemented conditions [35], [36].

To identify markers for TSC-enrichment in osteosarcoma, immunophenotyping for cell surface markers, transcriptome, and proteome analyses were performed. The absolute levels of various markers varied among the 3 cell lines. Further, heterogeneous expression of previously reported markers, such as nestin and CD133 [17], [37], was observed. The growing scientific evidence suggests that the transcript expression does not always correlate with the protein expression. In a recent study comparing RNA and protein expression profiles of 23 cell lines, significant correlations between the RNA and protein expression were observed for 33% of the gene products [38]. A variety of factors, such as posttranscriptional and posttranslational modifications, affect these outcomes. In our study, at the transcript level nestin expression is higher in CHA59 spheres than monolayers. This trend is maintained at the protein level for CHA59 cells. However, it is not observed for Saso-2 and HuO9 cells. Heterogeneous nestin expression is comparable to the variant or no nestin expression in different osteosarcoma patient samples, further supporting the tumor heterogeneity [37]. As for CD133, aside from heterogeneous expression, the absolute CD133 levels were negligible. Moreover, both CD133+ and CD133− melanoma cells have been reported to demonstrate comparable tumorigenicity in vivo [3], [39]. Our results highlight this fact – heterogeneity of tumors – and thus make a solid case for using multiple markers for identifying stem cells, as has been reported for other tumor types.

Despite these caveats, we identified some consistent expression patterns in spheres vs. monolayers. Lower CD326, CD24, CD44, and higher CBX3 and ABCG2 expression was observed in spheres as compared with monolayers in CHA59, Saos-2 and HuO9. A significant decrease in CD24 and CD44 expression accompanied sphere culture. CD24−CD44+ and CD24+CD44+ cells have been reported to possess TSC characteristics [14]. However, in a previous report by our group, we reported more TSC ability to recapitulate tumor populations in CD44− than CD44+ cells isolated from OVCAR-5 ovarian tumor cell line [27].

Consistently higher CBX3 protein expression in spheres as compared with monolayers was observed, indicating CBX3 as a marker for TSC-enrichment in osteosarcoma. CBX3, or heterochromatin protein 1 gamma, has been reported to function in chromatin packaging and gene expression regulation. It has been found to associate with nucleosomes in heterochromatin and possibly regulate euchromatin repression [26]. High CBX3 expression has been reported in myxoid liposarcoma, colon, breast, esophageal, cervical, and lung tumor patient samples [26].

ABCG2 has previously been reported to be associated with TSCs [33] including those derived from Saos-2 [17]. A significantly higher ABCG2 expression in CHA59, Saos-2, and HuO9 spheres than monolayers supports TSC-enrichment in spheres.

Significantly higher ABCA5 expression, a relatively newly discovered ABC transporter, in CHA59 and Saos-2 spheres vs. monolayers points to its utility as a putative TSC-enrichment marker in osteosarcoma. A recent study reported a significantly higher ABCA5 expression in Hoechst 33342-labeled side-population vs. non-side population cells in esophageal cancer [40]. ABCA5 was reported as a marker in prostate cancer biopsies and urine [41]. The drug substrate specificity of ABCA5 remains under investigation.

ALDH+ cells have been reported to possess higher tumorigenicity in a variety of tumors, such as breast, liver, and lung tumors [42], [43], [44], including osteosarcoma [11], though for osteosarcoma the enhanced tumorigenicity was achieved after serial passaging of Os 99-1 osteosarcoma cells in mice. Of note, ALDH positivity was found to be ineffective in selecting melanoma TSCs [45]. In a previous study, low ALDH expression was observed in Saos-2 and HuO9 cells [11]. We also observed the same trend, and measured comparable low ALDH expression in Saos-2 and HuO9 monolayers and spheres. We observed significantly higher ALDH+ cells in CHA59 spheres than monolayers. Further, we observed a distinct distribution of ABC transporters in ALDH+ vs. ALDH− cells. While ABCG2 was significantly higher in ALDH+ cells, ABCA5 was significantly higher in ALDH− cells. Importantly, while comparable POU5F1 and NANOG and significantly lower SOX2 transcripts were observed in CHA59 spheres as compared with monolayers, in the cells sorted on the basis of ALDH expression from spheres significantly higher POU5F1, NANOG, and SOX2 were observed in ALDH− as compared with ALDH+ cells. The observed ABC transporter and POU5F1, NANOG, and SOX2 expression indicates that ALDH− cells might have a distinct chemoresistance and stem cell phenotype from ALDH+ cells, and may play an as yet unidentified role in tumorigenesis.

Similar to heterogeneous gene and protein expression pattern, a heterogeneous drug sensitivity phenotype was observed for the 3 cells lines *in vitro.* Higher resistance to cisplatin has been reported in MG-63 spheres as compared with monolayers [6]. However, the study measured the drug sensitivity in whole spheres vs. monolayers, and the 3-dimensional structure of spheres might have contributed to the reported resistance. In contrast, we observed comparable resistance to cisplatin in both monolayers and spheres in CHA59, Saos-2, and HuO9. We performed the experiment with individual cells derived from spheres and monolayers. We observed higher drug resistance in spheres than monolayers against drugs, such as velcade, dasatinib, and sorefinib. Bioinformatic analysis (Figure S5B) demonstrated higher triple positive (ABCB1/ABCC1/ABCG2) cluster C2 population in Saos-2 spheres (17%) than monolayers (0%). In addition, higher ABCG2 positive cluster C3 in CHA59 spheres (14%) than monolayers (5%). Possibly, higher ABC transporter and ALDH expression in spheres corresponds with higher drug resistance in spheres as compared with monolayers.

Spheres showed higher sensitivity to 5-aza-C, vincristine, and RHPS4. Epigenetic alterations have been implicated in tumor formation. 5-aza-C, clinically approved to treat cancer patients, has been reported to inhibit DNMT1, 3a, and 3b in different tumors [46], [47]. Spheres demonstrated reduced resistance to 5-aza-C as compared with monolayers. This indicates an epigenetic sensitivity in spheres, and a possible targeted therapeutic opportunity against TSCs in osteosarcoma.

Rapidly dividing cells undergo cytoskeletal restructuring, such as formation of the mitotic spindle from tubulin. Vincristine inhibits tubulin polymerization, and thus kills rapidly proliferating tumor cells. Our results demonstrate higher sensitivity to vincristine in spheres as compared with monolayers.

Telomerase activity is crucial to maintain chromosomal integrity. Loss of telomerase activity in somatic cells limits their replicative potential and results in cellular senescence. On the other hand, stem cells retain telomerase activity. Rapidly replicating tumors and TSCs, have been reported to reacquire telomerase activity, and are thus a target for telomerase inhibitors, such as imetelstat, which is currently in clinical trials [48]. We observed higher sensitivity to RHPS4 in Saos-2 and HuO9 spheres as compared with monolayers.

The observed TSC marker heterogeneity is to be expected. For example, in a previously published report examining OCT3/4, SOX2, and NANOG expression in spheres from Saos-2, MG-63, HuO9, and Os 99-1, great variability was observed among the cell lines [5]. Despite this expected heterogeneity, our goal for the current study was to find molecules that are consistently expressed across an array of cell lines. In this regard, we observed significantly lower CD326, CD24, CD44 and higher ABCG2 transporter expression and CBX3 expression in TSC-enriched as compared with un-enriched cultures. More importantly, these consistent trends were observed across a panel of osteosarcoma cell lines: 1. CHA59 is from a 16-year-old Caucasian male, 2. Saos-2 is from an 11-year-old Caucasian female, and 3. HuO9 is from a 13-year-old Japanese female.

The marker expression data reported by us, and the heterogeneous nature of tumors argues against the expectation of finding pan-tumor markers. Further, a combination of epigenetic, genetic, and environmental effects result in variant expression patterns and distinct populations in the same tumor from different patients. Therefore, multiple markers are needed to identify TSCs in these different tumors. To conclude, enhanced clonogenicity, tumorigenicity, and drug sensitivity of spheres as compared with monolayers provide support for TSC-enrichment in spheres. Identification of ABCA5 and CBX3 in TSC-enriched spheres fulfilled our goal of finding osteosarcoma biomarker candidates that could be used in combination with already known markers, such as ABCG2 and ALDH. Further biological implications of CBX3 and ABCA5 need to be in independent studies using murine models and patient samples. We have identified vincristine, 5-Aza-C, and RHPS4 as potential therapeutic agents against TSC-enriched osteosarcoma cultures, which we hope will be tested in *in vivo* systems. The *in vivo* dependability of these markers needs to be confirmed in fresh patient samples. Lastly, our *in vitro* results suggest that 5-aza-C, vincristine, and RHPS4 could be tested *in vivo* for their potential for targeted TSC and tumor therapy in osteosarcoma.

## Materials and Methods

### Cell lines

CHA59 is freely available from the NCI, DTP Tumor Repository http://www.dtp.nci.nih.gov/branches/btb/tumor-catalog.pdf. Saos-2 cells were purchased from ATCC (VA, USA). HuO9 cells were purchased from Japanese Cancer Research Resources Bank, Tokyo, Japan. CHA59, Saos-2, and HuO9 were subjected to short tandem repeat analysis at the NCI core facility. The results (Figure S6) were interrogated in CLIMA (http://bioinformatics.istge.it/clima/index.php), which confirmed the uniqueness and contamination-free state of the 3 cell lines.

CHA59, Saos-2, and HuO9 monolayer cell cultures were maintained in RPMI-1640 (Lonza, USA) containing 10% fetal bovine serum (FBS) (Hyclone, USA) and 2 mM L-glutamine (Hyclone, USA). Spheres were cultured in RPMI-1640 containing 15% KnockOut Serum Replacement (Gibco, USA) (abbreviated as “SS” in the text), and 2 mM L-glutamine. Cultures were maintained in a 37°C humidified atmosphere with 5% CO_2_.

### Transmission electron microscopy

CHA59 monolayer cultures and spheres were washed two times in PBS (Invitrogen, USA), and treated with fixative solution (2% glutaraldehyde in 0.1 M cacodylate buffer), RT, 1 h. Spheres were centrifuged in a 15 ml conical centrifuge tube at ∼100 g, 10 min. The pellet was gently overlaid with fixative solution, RT, 2 h. After fixation, monolayer and sphere samples were postfixed in 1% osmium 1 h. They were rinsed in cacodylate buffer (0.1 M, pH 7.2), then in acetate buffer (0.1 M, pH 4.2) and stained in 0.5% uranyl acetate in the acetate buffer for 1 h. Cells were dehydrated in a series of graded alcohol washes followed by 100% propylene oxide. Subsequently, the samples were infiltrated in equal volumes of propylene oxide and epoxy resin (Poly/Bed 812, Polysciences, Inc. Warrington, PA). Cell monolayers were embedded in an epoxy resin, omitting propylene oxide wash and infiltration steps. The resin was cured in an oven set at 55°C for 48 h. Finally, ultrathin sections were made with an ultramicrotome (Ultracut UCT, Leica, USA.), mounted on a 200-mesh grid, stained with uranyl acetate and lead citrate (Leica, USA), stabilized by carbon evaporation (Edwards, UK), observed, and imaged by a digital camera (AMT, USA) at the desired magnifications with a transmission electron microscope (H7600, Hitachi, Japan) operated at 80 kV.

### Fast Red staining for alkaline phosphatase expression

Cells were plated at a density of 1.5×10^5^ per well in 6-well plates (Fisher Scientific, USA) and cultured until they reached 100% confluence (∼7 days). Subsequently, these were treated with osteogenic differentiation inducing cocktail containing 0.1 μM dexamethasone (Sigma, USA), 50 μm ascorbic acid (Sigma, USA), and 10 mM beta-glycerolphosphate (Sigma, USA) in RPMI 1640 containing 10% FBS and 2 mM L-glutamine every 2 days. Nine days post-treatment, cells were stained with Fast Red to detect ALPL expression.

### Transcriptome analysis

Total RNA was extracted from independent, triplicate samples of CHA59 monolayers and spheres using RNeasy Mini Kit (Qiagen, USA). These were arrayed on Affymetrix 133 plus 2 microarrays. The data was RMA normalized and uploaded to the website http://madb.nci.nih.gov/ maintained by the Advanced Technology Center at NCI/CCR. Data from each monolayer and sphere paired samples was compared and analyzed for genes modulated >2-fold in the sphere compared to the monolayer cells. These genes were interrogated using Ingenuity Pathways Analysis (IPA, www.ingenuity.com).

### Proteome analysis

#### Protein Preparation

CHA59 monolayer and sphere pellets were lysed with buffer containing 7 M urea, 2 M thiourea, 4% (W/V) CHAPS, 1% v/v Pharmalyte 3–10 (GE Biosciences, USA), 40 mM dithioerythritol [DTE], 50 mM Tris (pH 7.4) containing 1× protease inhibitor cocktail tablet. Cell lysates were sonicated twice for 10 sec and centrifuged to remove any insoluble material. Protein concentration was determined using Coomassie Plus reagent.

#### Two Dimensional Electrophoresis (2D-PAGE) and Image Analysis

For first dimension electrophoresis, a 400 µg protein sample was adjusted to 450 µL with rehydration buffer containing 8 M urea, 2% (m/v) CHAPS, 25 mM DTE, 1% v/v Pharmalyte 3–10 and 0.002% bromophenol blue, and applied to 24 cm Immobiline DryStrips (pH 3–10 NonLinear, Amersham GE Healthcare, USA). Rehydration and isoelectric focusing (IEF) were performed in the Ettan IPGphor apparatus (Amersham, GE Healthcare, USA) at 20°C, max. 80 µA per strip, according to the following program: 4 h at 0 V, 7 h at 30 V (rehydration); 1 h at 200 V, 1 h at 500 V, 1 h at 1000 V, then 8–12 h at 8000 V (IEF) until reaching 80–100 kVh. Subsequently, the IEF strips were cut into three equal pieces, equilibrated for 20 min with shaking in 5 mL of 50 mM Tris-HCl (pH 6.8), 6 M urea, 30% glycerol, 2% w/v SDS, and a trace of bromophenol blue containing 2% (w/v) DTE followed by 20 min in the solution containing 2.5% w/v iodoacetamide in place of 2% DTE. For the second dimension, the strips were placed on top of NuPAGE 4–12% Bis-Tris ZOOM mini gels (Invitrogen, USA), sealed with 0.5% agarose in NuPAGE MES SDS running buffer and run for 40 min at 200 V. The gels were washed with water, fixed in 7% acetic acid, 10% methanol for 30 min, and then stained overnight in SYPRO Ruby protein gel stain (Molecular Probes, USA). To decrease background fluorescence, the gels were destained in 7% acetic acid, 10% methanol for 30 min before imaging, using a Typhoon TRIO imager (GE Healthcare, USA) set to a resolution of 100 µm. The differentially expressed protein spots were visually examined.

#### Gel Cutting and In-gel Tryptic Digestion of Proteins

Protein spots were excised from the SYPRO Ruby stained gels and transferred to a 96-well plate. Gel spots were washed twice with 100 mM ammonium bicarbonate, dehydrated with acetonitrile and dried in a SpeedVac concentrator SC110A (Savant, Fisher Scientific, USA). The dry gel spots were rehydrated with 50 mM ammonium bicarbonate buffer containing 12.5 ng/µL sequencing grade porcine trypsin (Promega, USA) for 45 min on ice. The buffer was then removed, replaced with 50 mM ammonium bicarbonate and digestion carried out at 37°C for 16 h. The supernatant was removed and the tryptic peptides were extracted first with 25 mM ammonium bicarbonate and thereafter with 5% formic acid for 20 min each. The supernatant was pooled with the combined extracts, dried in a SpeedVac concentrator and dissolved in 30 µL 1% formic acid, 5% acetonitrile prior to mass spectrometric analysis of 10 µL aliquots.

#### Mass Spectrometry

The technique employed for protein identification involved microcapillary liquid chromatography – tandem mass spectrometry (LC – MS/MS). Trypsin digested protein samples were analyzed using a Finnigan LTQ ion trap mass spectrometer. CID spectra were analyzed using SEQUEST, against the Swiss-Prot indexed human protein database (Feb, 2009 release). The variable modification of +57.021 Da and +15.995 Da were set for iodoacetamide alkylated cysteinyl (Cys), and for oxidized methionine residues, respectively. A mass tolerance of 1.0 AMU was used for mass measurements in the MS mode and 1.5 AMU for the MS/MS mode. Only peptides possessing tryptic termini (allowing for up to two internal missed cleavages) possessing Δ correlation scores (CN) ≥0.1 and, ≥2.0 for [M + H]^1+^ peptides, ≥2.2 for [M + 2H]^2+^ peptides, and ≥3.5 for [M + 3H]^3+^ peptides, were considered legitimate identifications. Only proteins identified by at least two tryptic peptides excluding the same sequence with different charge states were considered. Each sample was analyzed twice. Out of the proteins with Xcorr values ≥20, VIME and CBX3 were selected for further analysis.

### Real-time RT-PCR

Total RNA was isolated using RNeasy Mini Kit (Qiagen, USA), and quantified using Nanodrop. Ten μg RNA was reverse transcribed using High Capacity cDNA Reverse Transcription Kit (Applied Biosystems, USA). One hundred ng cDNA was used per reaction to detect ALDH1A2, ABCA5, ABCG2, POU5F1, SOX2, NANOG, WNT1, WNT5B, NEUROD1, ETS1, PPARG, RUNX2, FOXA2, 18 s using TaqMan Gene Expression Assays (Applied Biosystems, USA). The data was normalized to 18 s and statistical significance was determined using a t-test in Excel.

### Flow cytometry for cell surface protein profiling

Flow cytometry for the 8 cell surface markers was performed as described in [27]. The samples were processed for 6-color (CD24∼PECy5, CD44∼Pacific Blue, CD133∼PE, CD166∼APC, CD326∼ Alexa700, and ABCB1∼FITC), 3-color (ABCB1∼FITC, ABCC1∼PE, and ABCG2∼ Alexa700), and 2-color (ABCC1∼ PE and ABCG2∼Alexa700) analyses. For antibodies and specific concentrations, see Figure S7. Isotype controls were used to set gates such that, for each cell, less than 1% of the total cell population was false-positive. Labeled cells were then analyzed (10,000 events). The data was analyzed for significance by the 2-tailed student t-test assuming equal variance in Microsoft Excel and p<0.05 was determined as significant.

### Cluster analysis and principal component analysis

Three biological replicates of CHA59 and Saos-2 monolayers and spheres were analyzed for 5 parameters – ABCB1, ABCC1, ABCG2, forward scatter (FSC), and side scatter (SSC) using 3-color flow cytometry. Over 100,000 data points, each representing a single cell as identified by flow cytometry, with values across the 5 parameters, were fed into the JMP8 software. *k-means clustering* distributed the data among 10 clusters. Subsequently, the values for each parameter in 10 clusters were averaged, and the value for each individual cluster was divided by the average to find variations from the average. Thereafter, a variation of <0.6 was considered below average (black background), 0.6–1.0 average (white background), and >1.0 above average (gray background). Next, the color coded clusters were channeled into 2 principal components and plotted in 2-dimensions.

### Aldehyde dehydrogenase detection

The ALDEFLUOR kit (Stem Cell Technologies, Canada) was used to identify cell populations with high ALDH enzymatic activity as per the manufacturer's instructions. Briefly, 1×10^6^ cells were resuspended in the assay buffer containing ALDH substrate. Negative control cells were incubated with ALDEFLUOR in the presence of the specific ALDH-inhibitor dimethylaminobenzaldehyde. After incubation for 30 min at 37°C, the cells were centrifuged, resuspended in the buffer, stained with 7-Amino-actinomycin D to discriminate viable cells from dead cells during the analysis on a FACSAria using Diva 5.0 (BD Biosciences, USA). ALDEFLUOR staining was detected in a green fluorescence channel, and the samples treated with DEAB were used to set the gates for the ALDH positive region. The readings (n = 3) were used to determine significance (p<0.05) using the 2-tailed student t-test assuming equal variance in Microsoft Excel.

### Western blotting

Monolayer and sphere whole cell protein lysates were prepared in RIPA buffer (Sigma, USA), containing Protease Inhibitor Cocktail Tablets (Roche Applied Science, USA), and anti-phosphatase PhosSTOP (Roche Applied Science, USA), according to the manufacturer's protocol. Briefly, monolayers were washed once with PBS and lysed in RIPA buffer. Spheres were centrifuged, supernatant was discarded, and the pellet was washed once with PBS and lysed in RIPA buffer. After incubation on ice for 5 minutes, the monolayer and sphere lysates were cleared by centrifugation at 10,000 rpm, 4°C, 10 min. The supernatant was stored at −80°C. The protein concentration of the lysates was measured using the BCA protein assay kit (Thermo Scientific, USA) according to the manufacturer's instructions. Subsequently, lysates were run on Novex® 4–20% Tris-Glycine Gels 1.5 mm, 10 well (Invitrogen, USA). Following this, protein bands were transferred from the gel to a PVDF membrane using iBlot (Invitrogen, USA). The membrane was treated sequentially with methanol, water, PBST, and blocking buffer (5% non fat dry milk in PBST). Subsequently, it was treated with primary antibodies against nestin (Abcam, USA), vimentin (Abcam, USA), CBX3 (Millipore, USA), and GAPDH (Biochain Institute, USA) in PBST. After incubation at 4°C, O/N, the membrane was washed 3 times in PBST. Afterwards, it was treated with HRP-conjugated secondary anti-mouse (Abcam, USA) or anti-rabbit (Rockland, USA) antibody, 1 h, RT. Finally, the membrane was washed 3 times in PBST, and bands were visualized using ECL kit (Amersham, USA) and GeneSnap (Syngene, USA) according to the manufacturer's protocol.

### Clonogenicity

Cells were disaggregated into single cells using TrypLE Express (Invitrogen, USA), and 20,000 and 50,000 CHA59, and 20,000 HuO9 cells were plated in SeaPlaque Agarose low melting (1% bottom layer; 0.4% top layer, Cambrex Bio Science Rockland, USA) containing 0.5X RPMI 1640 and either 10% FBS (FBS-A) or 15% SS (SS-A) and 2 mM L-Glutamine in 60 mm culture dishes (Corning, USA). These were incubated in a 37°C humidified atmosphere with 5% CO_2_. For CHA59, seeded at 20,000 cells/plate density in triplicate, colonies were stained with crystal violet, 5 fields per plate were imaged with a 10X objective, and ≥100 micron sized colonies were counted and statistically analyzed using a 2-tailed t-test with p≤0.05 as significant. Upon appearance of visible colonies in CHA59 (seeded at 50,000 cell/plate) and HuO9 (seeded at 20,000 cells/plate), photographic evidence was recorded using a phase contrast microscope with a 40X objective.

### Tumorigenicity

Cells were disaggregated into single cells using TrypLE Express (Invitrogen, USA). These single cell suspensions were diluted such that the indicated cell number was suspended in 100 μl of a 1∶1 mixture of culture medium and Matrigel (BD Biosciences, USA). Subsequently, cells were injected subcutaneously into the right axillary region of female NOD.SCID mice (Animal Production Program, NCI-Frederick, http://web.ncifcrf.gov) using 27G needles. Thereafter, tumor growth was assessed weekly with tumor mass calculated from bidimensional caliper measurements using the formula ([tumor length x tumor width)/2 =  tumor weight in mg. Masses that were >150 mg and grew progressively during the observation period (90 days) were defined as tumors.

### High-throughput drug sensitivity assay

CHA59, Saos-2, and HuO9 monolayers and spheres were disaggregated to single cells, and, using a TECAN preprogrammed robot, plated in 384-well plates in FBS and SS media respectively. To account for different proliferation rates in FBS and SS, the optimal numbers of monolayers and spheres that produced comparable cell proliferation in FBS and SS media over 4 days were determined. Because of this optimization, the difference in cell number measured at the end of the 4-day drug treatment reflected differential drug sensitivity and was not because of different proliferation rates in the media. The optimized cell numbers were treated with various small molecule inhibitors at 18 different concentrations, ranging from 7.6×10^−11^ M to 1×10^−5^ M to in triplicate. The wells were randomized to account for differential evaporation rates in the peripheral vs. middle wells in a 384-well plate. After 4 days of drug treatment, cell survival was measured by XTT assay [49] and recorded as IC50 in molar concentration. For the assay, IC50 was defined as the concentration of a small molecule required to produce 50% growth inhibition in a cell line relative to the control.

### Invasion assay in transwell

CHA59 monolayers and spheres were disaggregated to single cells and plated at a density of 50,000 or 75,000 cells per well in RPMI-1640 (Lonza, USA) containing 0.1% BSA in the upper chamber of matrigel-coated transwell filters (Costar, USA). The lower chamber was filled with either 0.1% BSA, 10% FBS, or 15% SS, and the invasion assay was carried out for 48 hours at 37°C in a humidified atmosphere with 5% CO_2_. Thereafter, cells sticking to the upper layes of the filters were scraped off, and the invaded cells at the bottom were stained with crystal violet, photographed with a 40X objective, and counted manually. Statistical significance was analyzed using a 2-tailed t-test assuming equal variance.

### High-throughput 7-day real-time kinetic migration and invasion assay

For invasion assay, the filter in the upper chamber of xCELLigence system (Roche, USA) was coated with matrigel. The lower chamber was filled with either 10% FBS or 15% SS. Monolayers and spheres were disaggregated to single cells and plated at a density of 1×10^5^ cells per well in RPMI-1640 (Lonza, USA) containing 0.1% BSA in the upper chamber. The kinetic data was recorded for ∼7 days as per the manufacturer's instructions. Statistical significance was analyzed using a 2-tailed t-test assuming unequal variance in Excel.

### CBX3 and ABCA5 transcript profiling in patient samples

To evaluate CBX3 and ABCA5 expression in patients, transcriptome data in the Gene Expression Omnibus (GEO) repository were analyzed [50]. Using accession number GSE14359, transcriptome profiles generated on Affymetrix Human Genome U133A Array for primary osteoblasts and osteosarcoma biopsies were accessed [51]. Among the available osteosarcoma samples, only those from patients up to 25 years of age were considered for further analysis. This age-based cutoff was selected because the extensive *in vitro* biomarker analysis was performed on cell lines derived from adolescents; therefore, when testing the relevance of these *in vitro* data in patients, age-related heterogeneity was minimized by limiting the analysis to young population. The selected samples were assigned to three groups: 1. Primary osteoblasts (one sample in duplicate), 2. Osteosarcoma (one grade 2 and three grade 3 biopsy samples in duplicate), and 3. Osteosarcoma metastasized to lung (one grade 3 biopsy sample in duplicate). For genes of interest, normalized linear mRNA hybridization signal intensities, available from GEO, were log2 transformed and p values were calculated using a 2-tailed t test assuming equal variance in MSExcel.

## Supporting Information

Figure S1 **Characterization of CHA59 osteosarcoma cells.** (A) CHA59 cells treated with osteoblastic differentiation cocktail and stained for alkaline phosphatase by Fast Red. (B) Osteoid production in CHA59 xenografts in NOD/SCID mice, visualized by H&E staining. (C) Karyotype of CHA59 cells. (D) Morphology of colonies formed by CHA59 and HuO9 monolayers and spheres in FBS-A and SS-A. Phase contrast images taken with a 40× objective. (E) Spheres possessed self-renewal ability. Phase contrast images taken with a 40× objective.
(TIF)

Figure S2 **Migration and invasion ability of spheres.** (A) CHA59 monolayers and spheres (seeded at 75,000 cells/well) demonstrated invasion ability towards the FBS chemoattractant in a 48-hour invasion assay, *p<0.05. (B) CHA59 spheres (seeded at 50,000 cells/well) demonstrated significantly higher invasion ability towards the FBS than the SS chemoattractant in a 48-hour invasion assay, *p<0.05. (C) HuO9 spheres showed significantly higher migration and invasion ability than monolayers towards the SS chemoattractant in a 7-day kinetic assay. (D) Migration kinetics of Saos-2 monolayers and spheres towards FBS and SS chemoattractants in a 7-day kinetic assay. (E) Saos-2 migrated cells harvested from the bottom chambers. Phase contrast images taken with a 40× objective.
(TIF)

Figure S3 **Two-dimensional PAGE analysis of CHA59 monolayer** (**top row**) **and spheres** (**bottom row**)**.** Vimentin (VIME), translationally controlled tumor protein (TCTP), malate dehydrogenase (MDHM), chromobox protein homolog 3 (CBX3), dihydropyrimidinase-related protein 2 (DPYL2), medium-chain specific acyl-CoA dehydrogenase (ACADM), fructose-bisphosphate aldolase C (ALDOC).
(TIF)

Figure S4 **ABCA5 expression in CHA59, Saos-2, and HuO9 monolayers and spheres measured by microarray analysis** (**A**) **or real-time RT-PCR** (**B, C**)**.** *p<0.05.
(TIF)

Figure S5 **Bioinformatics analysis of ABC transporter expression in CHA59 and Saos-2.** (A) CHA59 and Saos-2 monolayer and sphere ABC transporter immunophenotype grouped into 10 clusters. (B) Different distribution of populations in triple positive and single positive clusters in CHA59 and Saos-2 monolayers and spheres. (C) Principal Component Analysis distinguishes between CHA59 and Saos-2 cell, and between monolayers and spheres.
(TIF)

Figure S6 **Allelic distribution in the 16 target loci tested by STR analysis of CHA59, Saos-2, and HuO9 cell lines.**
(TIF)

Figure S7 **Antibody details for flow cytometry and/or FACS.**
(TIF)
