# Correction: Identification of CBX3 and ABCA5 as Putative Biomarkers for Tumor Stem Cells in Osteosarcoma

**DOI:** 10.1371/annotation/8c74aaee-897d-4682-b62d-d95a3506c210

**Published:** 2012-11-08

**Authors:** Vaibhav Saini, Curtis D. Hose, Anne Monks, Kunio Nagashima, Bingnan Han, Dianne L. Newton, Angelena Millione, Jalpa Shah, Melinda G. Hollingshead, Karen M. Hite, Mark W. Burkett, Rene M. Delosh, Thomas E. Silvers, Dominic A. Scudiero, Robert H. Shoemaker

During the production process, specific shading of Table 1 was lost. A correct version of Table 1 can be seen here: 

**Figure:**
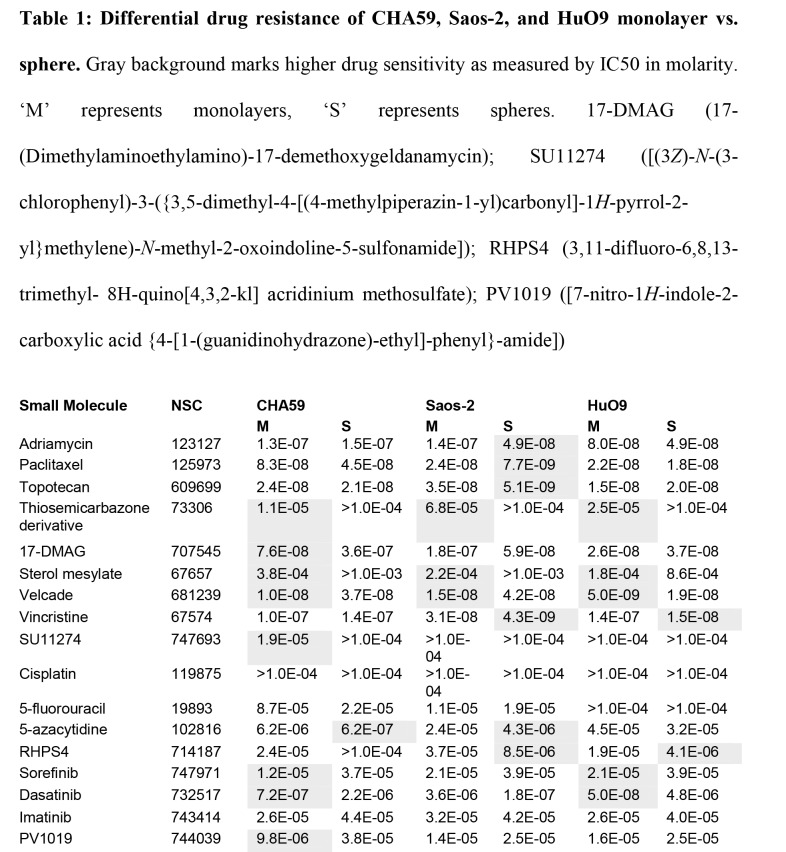


[^]

