# Supplementary figures and images for: Identification of CBX3 and ABCA5 as Putative Biomarkers for Tumor Stem Cells in Osteosarcoma
Source: PLoS One. 2012 Aug 3;7(8):e41401. doi: 10.1371/journal.pone.0041401 (PMC3411700; doi:10.1371/journal.pone.0041401)

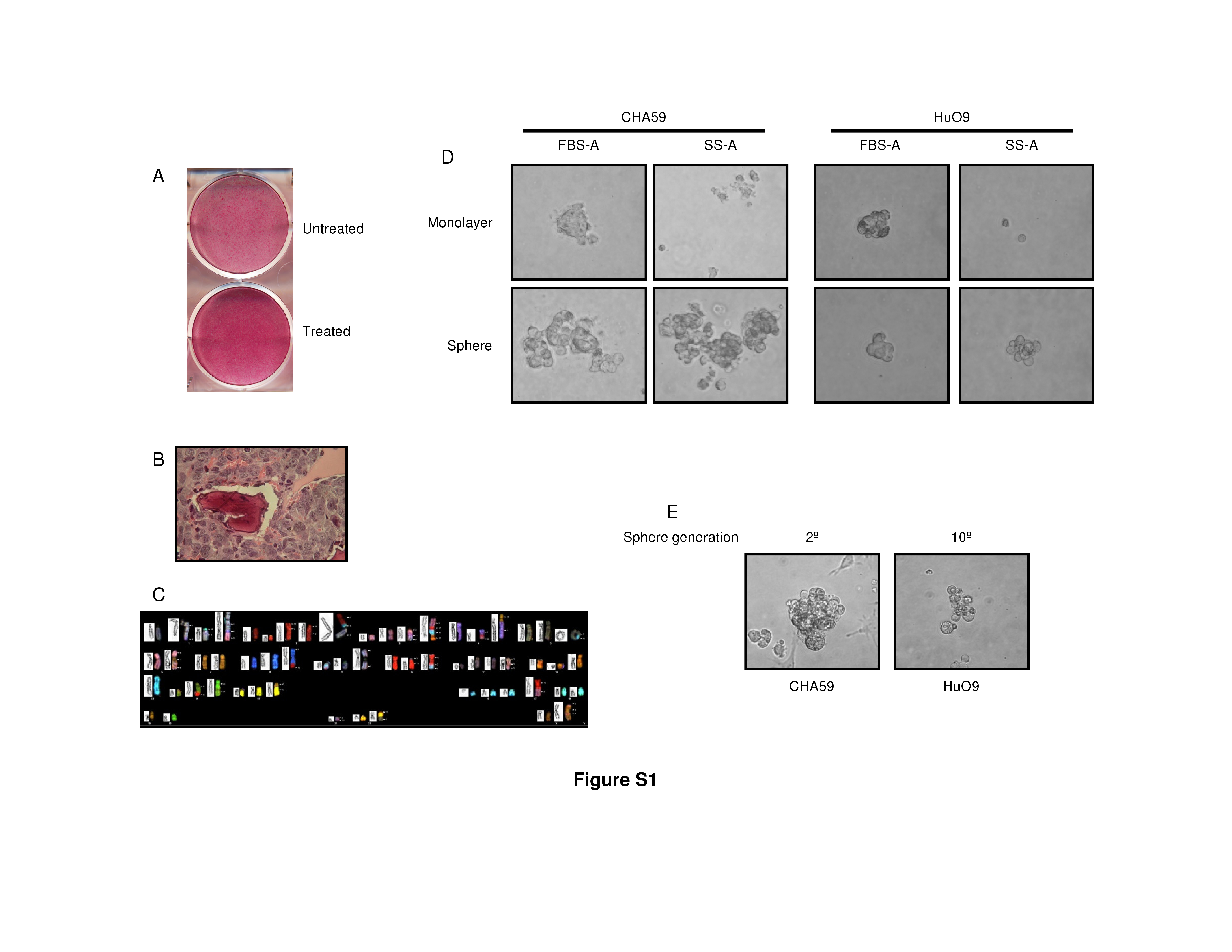

Supplement: Figure S1 — Characterization of CHA59 osteosarcoma cells. (A) CHA59 cells treated with osteoblastic differentiation cocktail and stained for alkaline phosphatase by Fast Red. (B) Osteoid production in CHA59 xenografts in NOD/SCID mice, visualized by H&E staining. (C) Karyotype of CHA59 cells. (D) Morphology of colonies formed by CHA59 and HuO9 monolayers and spheres in FBS-A and SS-A. Phase contrast images taken with a 40× objective. (E) Spheres possessed self-renewal ability. Phase contrast images taken with a 40× objective. (TIF) [file pone.0041401.s001.tif]

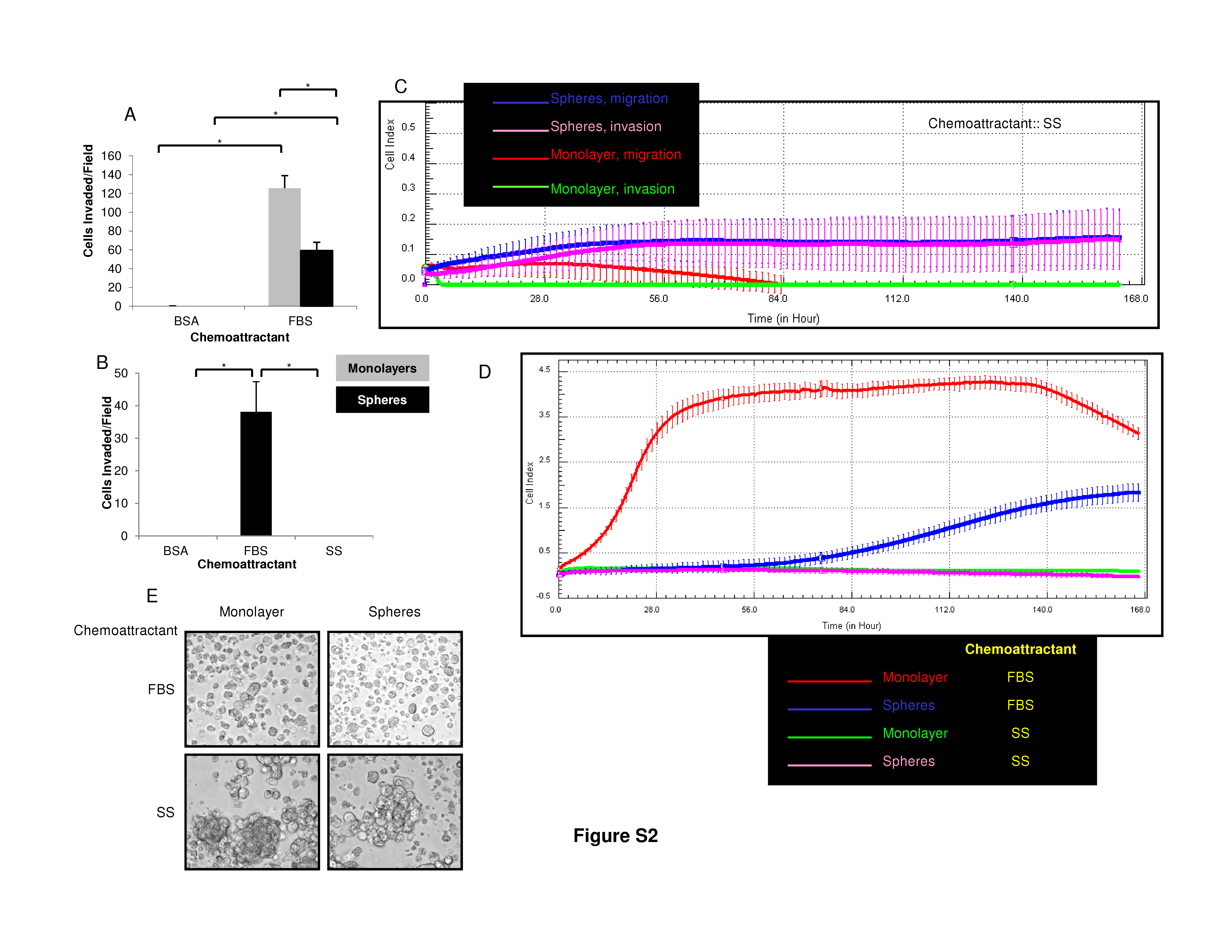

Supplement: Figure S2 — Migration and invasion ability of spheres. (A) CHA59 monolayers and spheres (seeded at 75,000 cells/well) demonstrated invasion ability towards the FBS chemoattractant in a 48-hour invasion assay, *p<0.05. (B) CHA59 spheres (seeded at 50,000 cells/well) demonstrated significantly higher invasion ability towards the FBS than the SS chemoattractant in a 48-hour invasion assay, *p<0.05. (C) HuO9 spheres showed significantly higher migration and invasion ability than monolayers towards the SS chemoattractant in a 7-day kinetic assay. (D) Migration kinetics of Saos-2 monolayers and spheres towards FBS and SS chemoattractants in a 7-day kinetic assay. (E) Saos-2 migrated cells harvested from the bottom chambers. Phase contrast images taken with a 40× objective. (TIF) [file pone.0041401.s002.tif]
